# Long-Term Cardiovascular Diseases of Heatstroke: A Delayed Pathophysiology Outcome

**DOI:** 10.7759/cureus.9595

**Published:** 2020-08-06

**Authors:** Farirai P Nzvere, Ezza Tariq, Katukuri Nishanth, Assam Arshid, Ivan Cancarevic

**Affiliations:** 1 Internal Medicine, California Institute of Behavioral Neurosciences & Psychology, Fairfield, USA; 2 Medicine, California Institute of Behavioral Neurosciences & Psychology, Fairfield, USA; 3 Medicine, Nishtar Medical College, Multan, PAK; 4 Surgery, California Institute of Behavioral Neurosciences & Psychology, Fairfield, USA

**Keywords:** heatstroke, heat-related illness, cardiovascular disease, ischemic heart disease, heart failure, heat stroke

## Abstract

Heatstroke, defined as an elevated core body temperature above 40°C accompanied by altered mental status (e.g., confusion, disorientation, seizure and coma), is the most severe and life-threatening condition in the spectrum of heat-related illnesses. Heatstroke patients may present with multi-organ dysfunction, but with rapid cooling and organ failure management, a full recovery often occurs within weeks. Long-term impairment is rare, with neurological impairment occurring most frequently. Despite an abundance of research on the persistent neurological and hepatic impairments, our knowledge of the long-term cardiovascular events in patients with heatstroke history is poor. We wondered whether heatstroke leads to cardiovascular diseases long after full recovery. Using Pubmed, Web of Science and Scopus, we gathered cohort studies looking at cardiovascular disease incidence or mortality as an outcome, including heatstroke animal studies. Based on the available literature, we found that a history of heatstroke is associated with an increased risk of cardiovascular diseases, including ischemic heart disease, heart failure and atrial fibrillation. Delayed metabolic disturbances occurring in exertional heatstroke mice are linked to the formation of atherosclerosis and the development of heart failure. These processes provide potential pathophysiological pathways leading to ischemic heart disease and heart failure in heatstroke patients. Our findings may massively impact our understanding of heatstroke recovery and the follow up of heatstroke patients. Therefore larger, more adequately powered cohort studies with cardiovascular disease as an outcome, in tandem with animal studies examining the underlying pathophysiology, are required to confirm or reject these findings and answer the proposed questions.

## Introduction and background

Increasing global temperatures, brought on by global climate change, are leading to an increased incidence of heat-related illnesses [1]⁠. Heat-related illnesses, an ancient range of extreme heat-related conditions, include heatstroke, heat exhaustion, heat cramps and heat rash [2]⁠. Heatstroke (HS), defined as an elevated core body temperature above 40°C accompanied by neurological dysfunction (e.g. confusion, disorientation, seizure and coma), is the most severe and life-threatening condition in the spectrum of heat-related illnesses [3]⁠. Although commonly associated with physical exertion, heatstroke is divided into two subtypes, 1) exertional heatstroke (EHS) and 2) classic non-exertional heatstroke (NEHS) [3]⁠. EHS often occurs in young adults participating in strenuous physical activities in hot environments, whereas NEHS commonly affects the elderly with underlying chronic health conditions or very young children [1]⁠. Both EHS and NEHS are preventable, and early diagnosis and rapid initiation of cooling can reduce mortality from approximately 80% to 10% [4,5]. Studies have shown that with rapid diagnosis and management, patients return to healthy organ function, as determined by clinical and biological markers of organ function [6]⁠. Altered sensorium (a sign of organ dysfunction) is the defining characteristic of heatstroke and differentiates it from heat exhaustion, which presents with hyperthermia without neurological dysfunction [3]⁠. Multiple organ dysfunction has been reported in heatstroke patients, leading Bouchama et al. to suggest an alternative HS definition, “It is a form of hyperthermia associated with a systemic inflammatory response leading to a syndrome of multi-organ dysfunction in which encephalopathy predominates” [3]. The combination of heat cytotoxicity and the body’s inflammatory and coagulation pathways response leads to multiple organ dysfunction, including encephalopathy, rhabdomyolysis, acute kidney injury, hepatic injury, circulatory failure, cardiac injury and disseminated intravascular coagulopathy (DIC) [3]⁠. In a recent review of persistent neurological impairment in HS patients, Lawton et al. found cerebellar dysfunction in 35% of survivors, consisting of cerebellar dysarthria and ataxia, with some patients in a coma for up to two years [7]⁠. A decrease in functional status at two years follow up, resulting in the need for elderly institution care, has also been reported in NEHS patients [8]⁠. These outcomes are in line with other studies that have also shown that the cerebellum is particularly susceptible to heat cytotoxicity leading to permanent damage [7,9]. Myocardial dysfunction in HS hospitalized patients has been reported, with elevated cardiac troponin levels representing myocardial damage [10-13]. The myocardial dysfunction has ranged from transient stress-induced cardiomyopathy with Takotsubo-like left ventricular dilation to ischaemic heart disease resulting from increased myocardial oxygen demand in the setting of reduced myocardial perfusion [11,12]⁠. With these cardiovascular dysfunction events and persistent neurological and hepatic impairments in mind, it raises the question of whether HS pathophysiology similarly presents with long-term cardiovascular diseases in HS patients. Unfortunately, few studies have looked into long-term cardiovascular events in patients with heatstroke history documented by previous hospitalization. Although advancing medical knowledge continues to lower in-hospital deaths and post-HS sequelae, the discovery of all possible sequelae will further improve HS outcomes [14]. So, determining whether a link between HS and subsequent cardiovascular disease (CVD) exists opens up discussions and optimizes healthcare provision in patients with heat-related illness history. Decision making in determining when it is safe to return to duty (in the military) or play (in athletes) after EHS also remains contentious [6]. Shedding light on underlying pathophysiological cardiac processes undetectable in current “Return To Play Heat Tolerance Testing” will optimize this decision-making process, and subsequent medical follow up [6,15]. In this literature review, we will aim to prove the association of heatstroke and delayed cardiovascular diseases, including a look at the possible underlying pathophysiology.

## Review

Methods and results

A literature search was conducted in Pubmed, Web of Science and Scopus from inception through June 2020. The search terms “heat stroke”, “heatstroke”, “heat illness” and “cardiovascular disease*”, “ischemic heart disease”, “heart failure”, “early death” were used, with no filters on sex, age, or language applied. Our keyword searches found 75 results in Pubmed, seven results in Web of Science and 237 results in Scopus. Titles and abstracts of all 319 results were independently reviewed for relevance, and a total of 315 research papers were excluded due to the removal of duplicates and lack of outcomes of interest, i.e., cardiovascular disease, ischemic heart disease (IHD), heart failure (HF) or early death. Three relevant human studies were included in this review (Table 1), in addition to one animal study investigating delayed myocardial metabolic dysfunction in mice post-EHS [16-19].

**Table 1:** Characteristics of Heatstroke Human Studies Included in Review HS: heatstroke; C: control; SD: standard deviation; N/A: not applicable. * Total follow up duration (mean follow up not available).

Study	Study Type	Sample size	Matched controls	Mean Follow-up duration (Years ± SD)	Number of Events (%)					
					All Cardiovascular Diseases		Ischemic Heart Disease		Heart Failure	
					HS	C	HS	C	HS	C
Wallace et al. [16]	Retrospective cohort	3,971	17,233	14.4 ± 7.2	25 (23)	128 (22)	8 (7)	35 (6)	N/A	N/A
Tseng et al. [17]	Retrospective cohort	628	1256	11.89± 13.32	N/A	N/A	194 (31)	186 (15)	N/A	N/A
Wang et al. [18]	Retrospective cohort	150	150	14*	49 (33)	25 (17)	15 (10)	10 (7)	5 (3)	3 (2)

Delayed cardiovascular diseases in heatstroke patients

**Figure 1:**
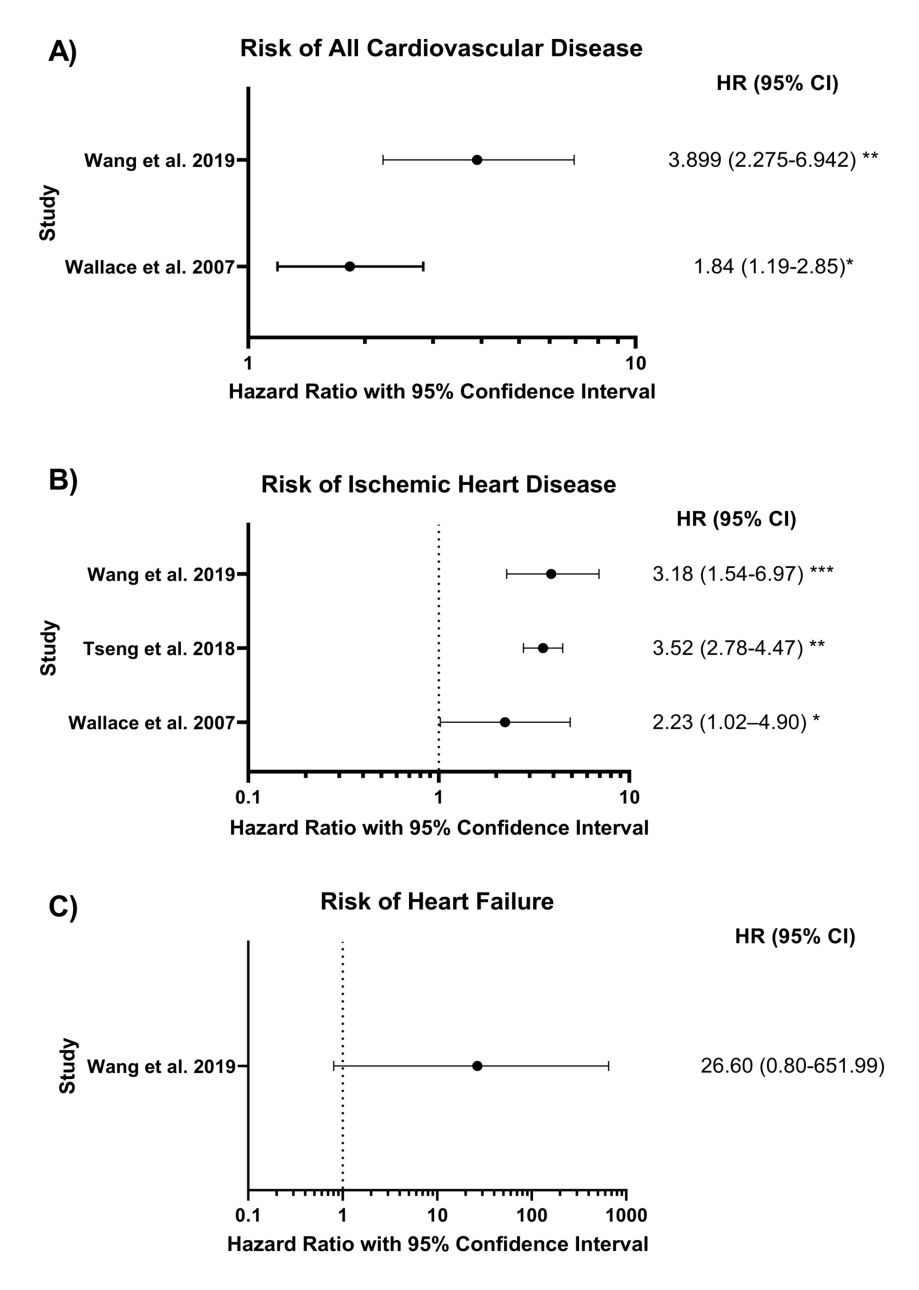
Risk of cardiovascular disease in heatstroke patients *P < 0.05, **P < 0.001, ***P = 0.001

**Figure 2:**
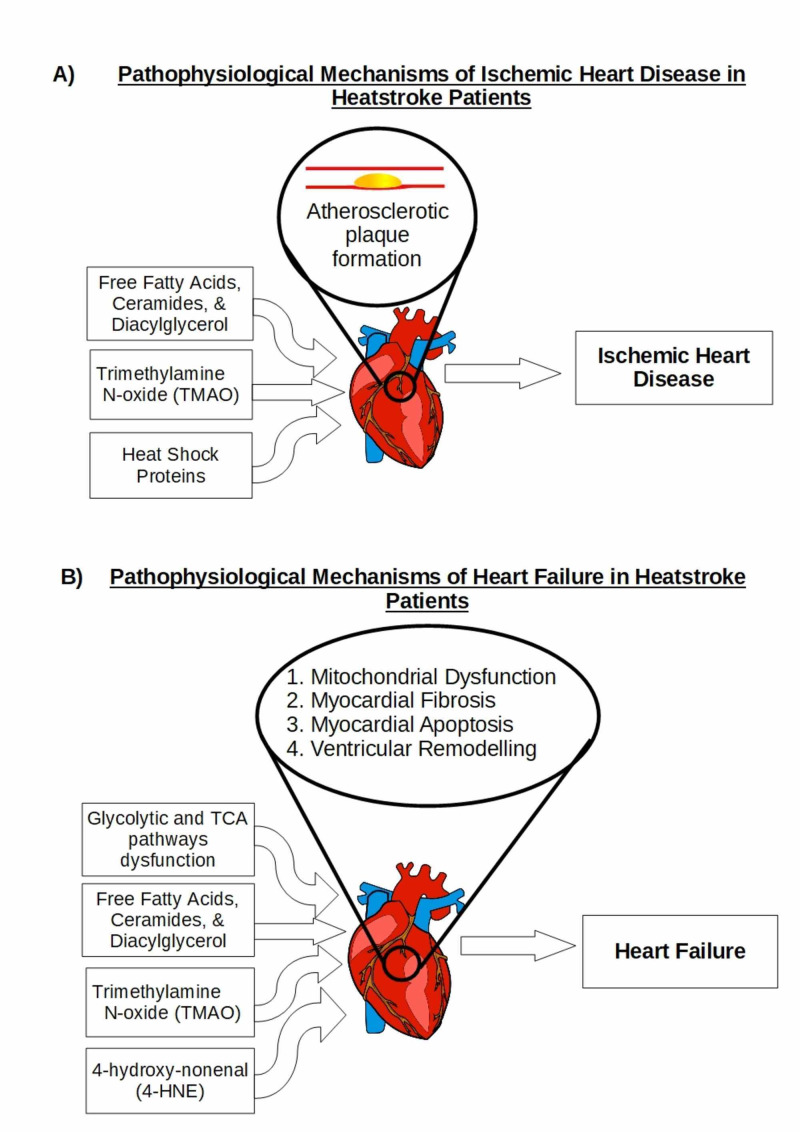
Potential pathophysiological mechanisms of cardiovascular diseases In heatstroke patients Note: Schematic showing the potential pathophysiological mechanisms of a) ischemic heart disease and b) heart failure in heatstroke patients.

Despite the full recovery of all organ functions within weeks, the cell damage done during a heatstroke (HS) event in the majority of heatstroke patients has the potential to induce disease later in life. This is especially true in the cardiovascular system. In the oldest and largest observational study, 3971 matched heatstroke patients were followed over 30 years [16]. Wallace et al. found that heatstroke patients had a 1.8 fold increased risk of all CVDs compared to matched non-heatstroke patients [16]. Wang et al. confirmed this association with a 3.9 fold increased risk of cardiovascular incidence in a cohort of patients followed for 14 years [18]. When compared to patients with other heat-related illnesses, heatstroke patients maintained a 1.5 fold increased risk of cardiovascular disease [17]. Furthermore, time to onset of CVD was 1.5 years earlier in the HS group than in the control group, and the average age of CVD onset or death was on average three years earlier in the HS group [16-18]⁠. Based on these study results, a history of heatstroke increases the risk of developing cardiovascular disease later in life. Ischemic heart disease and heart failure, the two most common CVDs in post-HS patients, share similar pathophysiological processes and ischemic heart disease is one of the leading causes of heart failure [20]⁠. Laitano et al. investigated the increased risk of subsequent CVD in this population in an animal study in which they found delayed metabolic dysfunction, suggesting that HS does indeed cause delayed onset of potential pathophysiological processes leading to cardiovascular dysfunction [19]. Laitano et al. discovered various metabolic disorders including 1) accumulation of fatty acids and their reactive metabolites, ceramide and diacylglycerol (DAG), 2) glycolytic and tricarboxylic acid (TCA) pathway disturbances, 3) increased oxidative stress, and 4) extensive inflammation in myocardial cells [19]⁠. Upon reviewing the literature, we found an increased risk of CVD in patients with a history of heatstroke, developing after initial recovery, as shown in Figure 1 [16-18]. Given the differences in incidence (Figure 1) and underlying pathophysiology (Figure 2) of IHD and heart failure, we will look at the two conditions separately.

Delayed ischemic heart disease in heatstroke patients

Ischemic heart disease, also known as coronary artery disease, is caused by an increased myocardial oxygen demand in the setting of reduced cardiac blood flow [21]⁠. Multi-organ failure during HS episodes can sometimes lead to ischemic heart disease in the form of ST-elevation myocardial infarction or stress-induced cardiomyopathy [10-12]. As discussed earlier, HS patients have a markedly elevated CVD risk. In a 30-year study of 3917 matched HS patients, Wallace et al. discovered a 2.3 fold increased risk of IHD in HS patients compared to their matched controls [16]⁠. Similarly, in more recent 13- and 14-year observational studies of HS patients, HS history increased the risk of developing IHD 3.5 fold (P < 0.001) and 3.1 fold (P = 0.003), respectively [17,18]. When looking specifically at acute myocardial infarction (AMI), HS-associated risk of AMI was slightly decreased at 2.7 fold compared to matched controls [18]. The studies mentioned above demonstrate that HS is associated with an elevated risk of IHD. An animal study revealing delayed metabolic dysfunction in EHS mice may explain the delayed onset of IHD [19]. Laitano et al. found evidence of global myocardial altered metabolism resulting in glycolytic and TCA pathway disturbances, accumulation of free fatty acids (FFAs) and their reactive metabolites, i.e. ceramides and DAG, and elevation of gut microbiota-derived trimethylamine N-oxide (TMAO) [19]. Accumulation of FFAs, ceramides and DAG, as a result of heat cytotoxicity and altered metabolism, are linked to mitochondrial dysfunction, cardiomyocyte lipotoxicity and endothelial dysfunction [19,22-25]⁠. These oxidative processes play a role in atherosclerotic plaque formation leading to IHD [24,25]. Another possible mechanism of IHD in EHS patients is the role of gut microbiota-derived metabolite TMAO [26-29]⁠. In the animal study, TMAO had a biphasic response, rising early (0.5 hours post-EHS) before returning to baseline at three days post-EHS and subsequently rising over the 14 days [19]⁠. Interestingly, in what is known as the “gastrointestinal paradigm of EHS”, studies have shown that EHS disrupts the gastrointestinal barrier, leading to leakage of gut microbiota and its accompanying metabolites into the circulatory system [26]⁠. TMAO, a gut microbiota-derived metabolite, not only increases platelet reactivity and thrombosis, but it is also involved in vascular endothelial dysfunction, resulting in atherosclerosis [26,27]. Elevated TMAO levels also have prognostic value in acute coronary artery syndrome (ACS) patients predicting short- and long-term ACS events [28,29]⁠. Lastly, heat shock proteins (HSPs), especially HSP70, are a group of molecular chaperones expressed in response to heat stress to protect against heat cytotoxicity and organ damage [30,31]. Unfortunately, overexpression of HSP70 has an anti-cytoprotective role and may result in pro-inflammatory and autoimmune responses which play a role in atherosclerosis [32].

An interesting secondary observation was the significantly higher risk of IHD in female HS patients (8.4 fold) compared to their male counterparts (2.9 fold) (P < 0.001) [17]⁠. Similar results were found in female mice in the later stages of recovery post-EHS, with significantly pronounced metabolic dysfunction and histological changes as compared to male mice post-EHS [19]. Whether this association is due to chance alone requires further investigation as other human observational studies have been heavily male-dominant [16,18]⁠. Upon reviewing the literature, we found an increased risk in the development of IHD post-HS, as shown in Figure 1B. The mechanisms mentioned above (Figure 2A) provide probable, but not definitive, IHD mechanisms to support the significantly increased risk of IHD in patients with a history of HS. The increased risk of developing ischemic heart disease in heatstroke patients warrants further evaluation with larger, higher power studies alongside animal models to further understand the underlying disease-causing mechanisms.

Delayed heart failure in heatstroke patients

Heart failure, the second most common CVD in HS patients, is considered a global pandemic due to its high incidence and high mortality [33]. Studies looking at HF as an independent endpoint are minimal. However, we found that in a 14-year follow up observational study, heart failure had a 26 fold increased risk in HS patients compared to matched controls (p = 0.06) (Figure 1C) [18]. This non-statistically significant result might be due to a small number of incidents. So an adequately powered study with a large enough sample size is needed to confirm the elevated risk statistically. Given the increased risk of all CVDs (3.9 fold increase), IHD (3.1 fold increase), AMI (2.7 fold increase), and atrial fibrillation (AF) (14.9 fold increase) in this study, there is probably an elevated risk of HF-maybe not at such a high value [18]. Cardiomyocytes exhibit limited proliferation capacity and cell turnover making the heart extremely vulnerable to oxidative stress. In an animal study on delayed myocardial metabolic dysfunction, Laitano et al. found evidence of altered myocardial metabolism resulting in glycolytic and TCA pathway disturbances, accumulation of FFAs and their reactive metabolites, i.e., ceramides and DAG, elevation of gut microbiota-derived TMAO, and other oxidative metabolites, e.g., 4-hydroxy-nonenal glutathione-a glutathione-adduct of 4-hydroxy-nonenal (4-HNE) [19]. Despite mostly returning to baseline levels by day three, the levels later rose significantly (day 9-14) [19]⁠. In addition, the myocardial tissue biopsy revealed key histological differences with increased signs of inflammation in the EHS hearts [19]⁠. Myocardial accumulation of FFAs, ceramides and DAG is not only linked to atherosclerotic plaque formation but is also involved in the development of myocardial fibrosis, apoptosis and decreased contractility leading to cardiac dysfunction [22,23,25,34-36]⁠. Ceramides have also been shown to induce insulin resistance: a leading etiological factor of diabetic cardiomyopathy [37]⁠. Glycolytic and TCA pathway disturbances documented in myocardial studies by Laitano et al. are a result of and a cause of mitochondrial dysfunction, implicated in cardiac dysfunction leading to HF [19,38]⁠. Recent evidence revealed that low concentrations of 4-HNE (a highly toxic oxidation product) have a cytoprotective effect. However, when exposed to elevated levels of aldehydes (e.g., 4-HNE), functional myocardial consequences ensue, resulting in HF [38]⁠.

Another possible mechanism of HF in HS patients is the role of gut microbiota-derived metabolite TMAO [26,39-41]. In the EHS animal study, TMAO had a biphasic response, rising early (0.5 hours post-EHS) before returning to baseline at three days post-EHS and subsequently rising later over the 14 days [19]⁠. As stated earlier, studies have shown that EHS disrupts the gastrointestinal barrier, leading to leakage of gut microbiota and its accompanying metabolites into the circulatory system [26]. Despite playing a proatherogenic role in IHD, elevated TMAO contributes to myocardial fibrosis, ventricular remodeling and dysfunction [39,40]⁠. Further, TMAO levels are strongly associated with major adverse cardiovascular events in HF patients [41]⁠. Finally, cardiovascular dysfunction is a common occurrence during an HS event [3]. Takotsubo-like stress-induced cardiomyopathy cases, one of the manifestations of cardiovascular dysfunction, have been reported in HS patients [12,13]⁠. However, despite complete resolution of this transient cardiomyopathy, myocardial fatty acid metabolism (which is severely impaired during the event) shows sustained impairment, and as noted earlier, FFA accumulation leads to the development of HF [11]⁠. Figure 2B summarises the pathophysiological pathways leading to HF in this population. The animal study alongside the non-statistically significant increased heart failure risk suggests that the risk is highly probable and not down to chance alone. However, the non-statistically significant increased heart failure risk warrants further studies to confirm or reject the risk.

The global picture

To our knowledge, this is the first review of its kind. Based on our findings, despite often presenting with acute cardiac dysfunction, heatstroke is associated with the development of cardiovascular diseases long after the initial clinical recovery phase. These findings then raise multiple questions: 1) How soon after a heatstroke event does the myocardial damage manifest clinically? 2) Is this occurrence of cardiovascular disease frequent enough to warrant post-EHS cardiac screening in this population? 3) Does rapid and effective cooling decrease the risk? and 4) Is further delaying the return to action necessary in athletes and military? As our planet’s temperature continues to rise, heat waves are becoming more frequent, leading to an increased incidence of heat-related illnesses in our vulnerable populations, namely, athletes, military, outdoor manual workers, elderly, and young children. Larger, more adequately powered prospective cohort studies with cardiovascular disease as an outcome, in tandem with animal studies examining the underlying pathophysiology are required to confirm or reject these findings and answer the proposed questions.

Limitations

Although the included studies excluded patients with previous cardiovascular disease and employed adjusted hazard ratios, our review exhibited some limitations. Firstly, only a small number of studies looking directly at long-term cardiovascular outcomes have been conducted. To counter this, our review included observational studies with large sample sizes followed over more than a decade. The animal study used to investigate potential pathophysiological pathways included mice observed for only 14 days post-EHS [19]. Compared to the onset of cardiovascular disease in our observational studies, 14 days is a relatively short period. However, at that period the mice had returned to regular activity, so when extrapolated to humans, this would coincide with the return to play (for athletes) and return to duty (for military) phase when HS patients are deemed clinically fully recovered [6,19]⁠. Another limitation of our study was the inclusion of retrospective cohort studies. As opposed to prospective studies, retrospective cohorts are potentially subject to unaccounted confounders such as health-related lifestyle factors and family history.

## Conclusions

Heatstroke, the most severe and life-threatening heat-related illness, often presents with multi-organ failure, returning to healthy organ function within days to weeks. In this review, we set out to understand whether heatstroke is associated with delayed cardiovascular disease occurring long after full recovery. We found that a history of heatstroke is associated with an increased risk of cardiovascular diseases including ischemic heart disease, heart failure and atrial fibrillation. Based on previous research, we know that certain delayed metabolic disturbances occurring in EHS mice are linked to the formation of atherosclerosis and the development of heart failure. These processes provide potential pathophysiological pathways leading to the development of ischemic heart disease and heart failure in this population. These findings may massively impact our understanding of heatstroke recovery and the follow up of heatstroke patients. Therefore, larger, more adequately powered prospective cohort studies with cardiovascular disease as an outcome, in tandem with animal studies examining the underlying pathophysiology, are required to confirm or reject these findings and answer the proposed questions.

## References

[1] (2020). Information and public health advice: heat and health. Who.

[2] Drake DK, Nettina SM (1994). Recognition and management of heat-related illness. Nurse Pract.

[3] Bouchama A, Knochel JP (2002). Heat stroke. N Engl J Med.

[4] (2020). Heat stroke. https://emedicine.medscape.com/article/166320-overview#a2.

[5] Walter E, Steel K (2018). Management of exertional heat stroke: a practical update for primary care physicians. Br J Gen Pract.

[6] OʼConnor FG, Heled Y, Deuster PA (2018). Exertional heat stroke, the return to play decision, and the role of heat tolerance testing. Curr Sports Med Rep.

[7] Lawton EM, Pearce H, Gabb GM (2019). Review article: environmental heatstroke and long‐term clinical neurological outcomes: a literature review of case reports and case series 2000-2016. Emerg Med Australas.

[8] Argaud L, Ferry T, Le QH (2007). Short- and long-term outcomes of heatstroke following the 2003 heat wave in Lyon, France. Arch Intern Med.

[9] Yaqub BA (1987). Neurologic manifestations of heatstroke at the Mecca pilgrimage. Neurology.

[10] Hausfater P, Doumenc B, Chopin S (2010). Elevation of cardiac troponin I during non-exertional heat-related illnesses in the context of a heatwave. Crit Care.

[11] Kurisu S, Inoue I, Kawagoe T (2003). Myocardial perfusion and fatty acid metabolism in patients with tako-tsubo-like left ventricular dysfunction. J Am Coll Cardiol.

[12] Chen WT, Lin CH, Hsieh MH, Huang CY, Yeh JS (2012). Stress-induced cardiomyopathy caused by heat stroke. Ann Emerg Med.

[13] Wakino S, Hori S, Mimura T, Satoru M, Fujishima S, Aikawa N (2005). A case of severe heat stroke with abnormal cardiac findings. Int Heart J.

[14] McDermott BP, Casa DJ, Ganio MS, Lopez RM, Yeargin SW, Armstrong LE, Maresh CM (2009). Acute whole-body cooling for exercise-induced hyperthermia: a systematic review. J Athl Train.

[15] Asplund CA, O’Connor FG (2016). Challenging return to play decisions: heat stroke, exertional rhabdomyolysis, and exertional collapse associated with sickle cell trait. Sports Health.

[16] Wallace RF, Kriebel D, Punnett L, Wegman DH, Amoroso PJ (2007). Prior heat illness hospitalization and risk of early death. Environ Res.

[17] Tseng MF, Chou CL, Chung CH, Chien WC, Chen YK, Yang HC, Chu P (2019). Association between heat stroke and ischemic heart disease: a national longitudinal cohort study in Taiwan. Eur J Intern Med.

[18] Wang JC, Chien WC, Chu P, Chung CH, Lin CY, Tsai SH (2019). The association between heat stroke and subsequent cardiovascular diseases. PLoS One.

[19] Laitano O, Garcia CK, Mattingly AJ (2020). Delayed metabolic dysfunction in myocardium following exertional heat stroke in mice. J Physiol.

[20] Cleland JG, McGowan J (1999). Heart failure due to ischaemic heart disease: epidemiology, pathophysiology and progression. J Cardiovasc Pharmacol.

[21] NHLBI NHLBI (2020). Coronary heart disease. Natl Hear Lung, Blood Inst.

[22] Boudina S, Sena S, Theobald H (2007). Mitochondrial energetics in the heart in obesity-related diabetes: direct evidence for increased uncoupled respiration and activation of uncoupling proteins. Diabetes.

[23] Law BA, Liao X, Moore KS (2018). Lipotoxic very-long-chain ceramides cause mitochondrial dysfunction, oxidative stress, and cell death in cardiomyocytes. FASEB J.

[24] Symons JD, Abel ED (2013). Lipotoxicity contributes to endothelial dysfunction: a focus on the contribution from ceramide. Rev Endocr Metab Disord.

[25] Schulze PC, Drosatos K, Goldberg IJ (2016). Lipid use and misuse by the heart. Circ Res.

[26] Chioncel O, Ambrosy AP (2019). Trimethylamine N‐oxide and risk of heart failure progression: marker or mediator of disease. Eur J Heart Fail.

[27] Wang Z, Klipfell E, Bennett BJ (2011). Gut flora metabolism of phosphatidylcholine promotes cardiovascular disease. Nature.

[28] Gencer B, Li XS, Gurmu Y (2020). Gut microbiota‐dependent trimethylamine N‐oxide and cardiovascular outcomes in patients with prior myocardial infarction: a nested case control study from the PEGASUS‐TIMI 54 trial. J Am Heart Assoc.

[29] Li XS, Obeid S, Wang Z (2019). Trimethyllysine, a trimethylamine N-oxide precursor, provides near- and long-term prognostic value in patients presenting with acute coronary syndromes. Eur Heart J.

[30] Lam KK, Cheng PY, Lee YM, Liu YP, Ding C, Liu WH, Yen MH (2013). The role of heat shock protein 70 in the protective effect of YC-1 on heat stroke rats. Eur J Pharmacol.

[31] Wang JL, Ke DS, Lin MT (2005). Heat shock pretreatment may protect against heatstroke-induced circulatory shock and cerebral ischemia by reducing oxidative stress and energy depletion. Shock.

[32] Xu Q (2002). Role of heat shock proteins in atherosclerosis. Arterioscler Thromb Vasc Biol.

[33] Savarese G, Lund LH (2017). Global public health burden of heart failure. Card Fail Rev.

[34] Chokshi A, Drosatos K, Cheema FH (2012). Ventricular assist device implantation corrects myocardial lipotoxicity, reverses insulin resistance, and normalizes cardiac metabolism in patients with advanced heart failure. Circulation.

[35] Chiu HC, Kovacs A, Ford DA (2001). A novel mouse model of lipotoxic cardiomyopathy. J Clin Invest.

[36] Basu R, Oudit GY, Wang X, Zhang L, Ussher JR, Lopaschuk GD, Kassiri Z (2009). Type 1 diabetic cardiomyopathy in the Akita (Ins2WT/C96Y) mouse model is characterized by lipotoxicity and diastolic dysfunction with preserved systolic function. Am J Physiol Heart Circ Physiol.

[37] Holland WL, Brozinick JT, Wang LP (2007). Inhibition of ceramide synthesis ameliorates glucocorticoid-, saturated-fat-, and obesity-induced insulin resistance. Cell Metab.

[38] Anderson EJ, Katunga LA, Willis MS (2012). Mitochondria as a source and target of lipid peroxidation products in healthy and diseased heart. Clin Exp Pharmacol Physiol.

[39] Xu H, Wang X, Feng W, Liu Q, Zhou S, Liu Q, Cai L (2020). The gut microbiota and its interactions with cardiovascular disease. Microb Biotechnol.

[40] Organ CL, Otsuka H, Bhushan S (2016). Choline diet and its gut microbe-derived metabolite, trimethylamine n-oxide, exacerbate pressure overload-induced heart failure. Circ Hear Fail.

[41] Suzuki T, Yazaki Y, Voors AA (2019). Association with outcomes and response to treatment of trimethylamine N‐oxide in heart failure: results from BIOSTAT‐CHF. Eur J Heart Fail.

